# Risk Analysis and Technology Assessment of Emerging (Gd,Ce)_2_O_2_S Multifunctional Nanoparticles: An Attempt for Early Safer-by-Design Approach

**DOI:** 10.3390/nano12030422

**Published:** 2022-01-27

**Authors:** Anh-Minh Nguyen, Ana Elena Pradas del Real, Olivier Durupthy, Sophie Lanone, Corinne Chanéac, Sophie Carenco

**Affiliations:** 1 Laboratoire de Chimie de la Matière Condensée de Paris, Collège de France, Sorbonne Université, CNRS, 4 Place Jussieu, 75252 Paris, France; anhminh.nguyen@nexdot.fr (A.-M.N.); olivier.durupthy@sorbonne-universite.fr (O.D.); 2 INSERM, IMRB, University Paris Est Creteil, 94010 Creteil, France; 3 ESRF, 71 Avenue des Martyrs, 38000 Grenoble, France; ana.elena.pradas@madrid.org

**Keywords:** safer-by-design, nanoparticles, oxysulfides, lanthanides, ROS, cerium, murine macrophage RAW 264.7

## Abstract

Acceptability and relevance of nanoparticles in the society is greatly improved using a safer-by-design strategy. However, this is difficult to implement when too late in the development process or when nanoparticles are already on the market (e.g., TiO_2_). We employ this strategy for emerging nanoparticles of lanthanide oxysulfide of formula (Gd,Ce)_2_O_2_S, relevant for photocatalysis as well as for multimodal imaging, as the bandgap of the nanoparticles, related to their Ce content, impacts their ability to absorb visible light. As a first step, we investigated the production of reactive oxygen species (ROS) as a function of cerium content, in abiotic conditions and in vitro using murine macrophage RAW 264.7 cell line. We demonstrate that, at sub-lethal doses, Ce-containing oxysulfide nanoparticles are responsible for a higher ROS intracellular formation than cerium-free Gd_2_O_2_S nanoparticles, although no significant inflammatory response or oxidative stress was measured. Moreover, there was no significant loss of cerium as free cation from the nanoparticles, as evidenced by X-ray fluorescence mapping. Based on these results, we propose a risk analysis for lanthanide oxysulfide nanoparticles, leading to a technology assessment that fulfills the safer-by-design strategy.

## 1. Introduction

Nanomaterials present fascinating properties that have made them highly attractive for novel marketable products or nanotechnology development as well as innovative academic research project. Consequently, the world production of nanomaterials continues to increase mainly in seven sectors of activity, which are paints and coatings, adhesives, energy, wellness and cosmetics, health, electronics and consumer goods [1]. In research and development, efforts are continuing to develop novel nanomaterials to further improve efficiency, to bring new properties and functionalities or to reduce cost. Despite the bright outlooks for the future of nanotechnology, nanomaterials carry potential risks towards environment and human health; the toxicity of nanomaterials can be higher than their chemically identical bulk counterpart, which limits the potential for innovation in both industry and research [2,3,4]. Due to their small size, nanoparticles are not only chemically more reactive, but they may also enter inside the cells and cause irreparable damage precluded from larger particles [5]. Several examples are available, such as pulmonary inflammation due to nanoscale TiO_2_ particles [3,6,7] and genotoxicity from Ag, ZnO [2,4,8,9] or carbon nanotubes [2,8,10,11]. Systematic evaluation of nanomaterial toxicity is still challenging. While it is well established that toxicity can be directly correlated with the release of toxic ions by dissolving nanomaterials as in the case of quantum dots containing cadmium, mercury or lead, for example, other more complex phenomena may also be incriminated [12,13]. Indeed, for non-dissolving nanomaterials such as TiO_2_, their bandgap and conduction band energy levels [14,15] have been connected to their potential toxicity through oxidative stress [4,16] in a multi-step process involving reactive oxygen species (ROS) production [16]. However, as of now, no clear and direct relationship can be established between nanomaterial toxicity and their physicochemical characteristics in a more general way.

In order to address the potential harms of nanomaterials while exploiting their ability to respond to economic and social issues, safer-by-design concept (SbD) has been introduced and developed to reduce to an acceptable level for society, the uncertainties related to inherent human and environmental risks of industrial innovation [17,18,19]. It consists of integrating knowledge of potential adverse effects into the process of designing manufactured materials at early stage of the life cycle, from their synthesis to their integration in functional products, their use, end-of-life and recycling. This concept was primarily employed for existing well-known nanomaterials after market introduction, in an incremental and progressive manner. A recent study on sunscreens gives a list of recommendations for a safer formulation of these cosmetic products and suggests a stronger complexation of surface atoms to reduce dissolution and ROS production [20,21].

Another strategy is to change the composition of the inorganic core rather than the surface shell to modify its interaction with the biological medium. Iron doping of ZnO and CuO nanoparticles limits both the release by dissolution of Zn^2+^ and Cu^2+^ ions in the medium and the oxidative stress toxicity [22,23,24]. In the same way, doping TiO_2_ nanoparticles with Fe^3+^ to reduce the bandgap, hence increasing the absorption of visible light [25]. However, this later example resulted in higher oxidative stress and cell death to macrophages-like RAW 264.7 cells. This highlighted the relevance of integrated effort of nanomaterials synthesis and safety assessment in safer-by-design strategies.

The question is to know how academic research can integrate new practices and tools to apply “Safer by design” at the lower technology readiness level (TRL 1–3) rather than at posteriori of the innovation process and before the integration of nanomaterials in high-tech products. Van Wezel and al. propose a set of easy-to-answer questions regarding risk analysis and technology assessment (RATA) as safe innovation tool to promote a responsible research and innovation (RRI) for the development of emerging technologies [26]. These questions to check the RATA awareness are addressed to all innovation stakeholders from lower TRL to higher, and answers will allow building a common database that will be enriched step by step along the innovation chain. The questions were developed by NanoNextNL which is a consortium of the government of the Netherlands including 130 companies, universities, knowledge institutes, and university medical centers, which is aimed at research into micro- and nanotechnology. They range from market opportunities to legislative aspects, hazards and fate of products, to possible pathways of emission of nanomaterials and mitigation strategies to limit emissions. In parallel, issues relating to the stakeholders involved, their issues, responsibilities, obligations and mutual relations as well as issues relating to societal consequences cover the technological development part. All these issues are very broad, and some will only concern certain categories of actors. 

L.G. Soeteman-Hernandez et al. applied this SbD concept with PhD students by taking case studies of nanomaterials from current research and published a first database addressing nanosafety aspects for nanomaterials as an example to raise awareness of the importance of risk assessment in the early stages of research and to implement RATA to academic laboratories [27]. 

In the future, these databases should be used to make decisions throughout the design stages of a nanotechnology-based product in a circular economy approach. They will make it possible to find the best compromise between the lowest inherent toxicity and the highest functionality, to define ways of applying the material and using the product to limit their emission or, in the least favorable cases, to establish minimizing measures to prevent undesirable effects right from the product conception.

In the present study, we investigate emerging multifunctional nanoparticles of pristine gadolinium oxysulfide Gd_2_O_2_S and Ce-doped (Gd,Ce)_2_O_2_S still far from the market, in the spirit of RATA as part of an ethical and civic approach. Gadolinium oxysulfide (Gd_2_O_2_S) nanoparticles are promising candidates for biotechnologies as they have been used as MRI contrast agent or X-ray absorbing materials. When doped with cerium, they gain an additional functionality as visible light absorber due to the decrease in the material bandgap from 4.7 to 2.1 eV. Further biomedical applications may be envisioned thanks to the combined antioxidant properties of cerium and the magnetic properties of gadolinium [28]. In contrast to most lanthanides which are trivalent, cerium can be both trivalent Ce^III^ and tetravalent Ce^IV^. Thus, the presence of cerium in the material requires special attention, because of the potential toxicity of each cation, as well as the modified properties of the materials in itself (e.g., surface charge). At the same time, the Ce^3+^/Ce^4+^ redox couple is responsible for the catalytic and antioxidant properties of cerium(IV) oxide CeO_2_ nanoparticles [28,29,30]. Here, we investigate both pristine Gd_2_O_2_S and Gd_2_O_2_S doped with different content of cerium, prepared from a similar synthesis route [31,32], and we analyze few indicators relevant to the toxicity under visible light in a risk analysis approach.

For this purpose, the ROS production of (Gd,Ce)_2_O_2_S nanoparticles was investigated. Experiments in abiotic conditions were performed with five samples containing Ce:Gd ratio from 0 to 50%. Ce-containing nanoparticles produced more ROS than Gd_2_O_2_S nanoparticles, both in the dark and under irradiation with 501 nm light. In vitro experiments using murine macrophage RAW 264.7 cell line were then performed. Several end-points were explored, such as mitochondrial activity, oxidative stress and inflammatory response at lethal and sub-lethal nanoparticle doses. A significant increase in intracellular ROS production was observed for macrophages incubated with Ce-containing nanoparticles. X-ray fluorescence mapping was employed to demonstrate that there is no significant loss of cerium by the nanoparticles in vitro. Finally, all the results were used to build a risk analysis and technology assessment table for these materials based on an appropriate selection of the set of questions proposed by van Wezel to evaluate the safety of these materials [26].

## 2. Results and Discussion

### 2.1. Gadolinium-Cerium Oxysulfide Nanoparticles

#### 2.1.1. Synthesis and Structure

Gd_2_O_2_S and (Gd,Ce)_2_O_2_S nanoplatelets were prepared following a protocol described previously and detailed in ESI [31]. Briefly, Gd(acac)_3_∙*x*H_2_O and Ce(acac)_3_∙*x*H_2_O) (acac = acetylacetonate) were used as metal precursors in relative amount corresponding to the expected final stoichiometry and reacted at 310 °C for 30 min with elemental sulfur (S_8_) in the presence of sodium oleate [33]. The metal and sulfur sources were added to a mixture of organic compounds containing oleic acid (OA), oleylamine (OAm) and 1-octadecene (ODE) in large excess, which played the roles of surface ligands and solvents. At the end of the synthesis, the nanoparticles were centrifuged and washed with ethanol and *n*-hexane. The final powder is composed of the nanoplatelets surrounded by oleate ligands [34]. 

**Figure 1:**
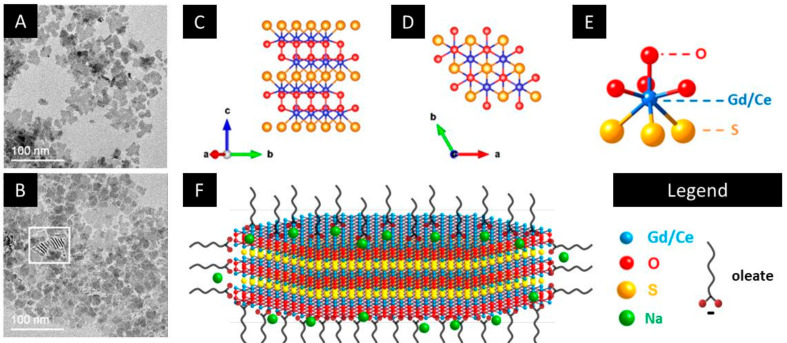
Transmission electron microscopy of Gd_2_O_2_S (**A**) and GdCeO_2_S (**B**) nanoparticles. In (**B**), the white square indicates nanoparticles that are staked and observed sideways. (**C**) Representation of the lamellar cristallographic structure of Gd_2_O_2_S. (**D**) View from the (001) direction of the structure. (**E**) Environment of Gd/Ce in the structure. (**F**) Schematic representation of [Ln_2_O_2_]^2+^-terminated Gd_2(1−*x*)_Ce_2*x*_O_2_S nanoparticles covered with oleate ligands.

The size and the shape of the Gd_2_O_2_S and GdCeO_2_S nanoparticles were characterized by transmission electron microscopy (TEM) (Figure 1A,B). The nanoparticles feature anisotropic 2D morphology and are highly stacked. As a result, precise measurement of the dimension of their basal facets remains desirable. For all nanoparticles samples, it is roughly estimated to be 20 ± 5 nm. On the other hand, the thickness can be determined thanks to stacked nanoplatelets that expose their side facets. Nanoplatelets are 2 ± 1 nm thick. The results indicate no significant difference in size and shape between monometallic gadolinium oxysulfide nanoparticles and bimetallic gadolinium-cerium oxysulfide nanoparticles.

Within the nanoparticles, cerium is located in the lanthanide site of the Ln_2_O_2_S structure (Figure 1C–E), and forms a solid solution with gadolinium, meaning that the Gd and Ce ions are statistically distributed amongst the crystallographic sites, as discussed in a previous study, which also shows the X-ray diffraction pattern of the nanoparticles powder [31]. It is bond to oxygen and sulfur in the structure, with iono-covalent bonds. Its formal oxidation state is Ce^III^ although air exposure of the nanoparticle powder results in partial oxidation to Ce^IV^ [31]. The nanoparticles can be schematized as 2 nm thick nanoplatelets covered with oleates ligands (Figure 1F) [34]. A detectable amount of sodium, which was introduced during the colloidal synthesis, is present in the powder (see Table S1). Indeed, the sodium cations are expected to facilitate the crystallization of the lanthanide oxysulfide compounds, but do not get incorporated into the inorganic core of the nanoparticles [33].

#### 2.1.2. Light Absorption and Optical Bandgap

**Figure 2:**
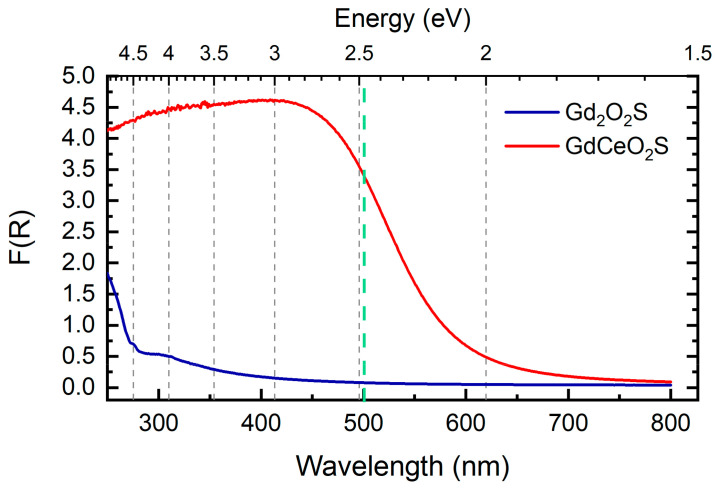
UV-visible diffuse reflectance spectra of Gd_2_O_2_S and GdCeO_2_S nanoplatelets. The *y*-axis is expressed as the Kubelka-Munk function F(R) calculated from obtained apparent absorbance. The dashed line indicates the wavelength at which the following ROS production experiments were carried out.

Absorption spectra of the powders of the nanoplatelets were recorded using UV-visible diffuse reflectance spectroscopy (Figure 2). The Kubelka–Munk function of reflectance F(R) was calculated from obtained apparent absorbance. This function is directly proportional to the extinction coefficient of the material, thus to its absorption properties. The data were fitted assuming a direct bandgap, as discussed in a previous study [35]. 

The Ce/Gd substitution strongly affects the absorption of the nanomaterials, in accordance with their color. In fact, spectrum of the synthesized Gd_2_O_2_S nanoparticles indicates only weak UV absorption with a threshold around 270 nm. The bandgap of the material was previously estimated at ca. 4.7 eV using the Tauc plot suggesting that Gd_2_O_2_S is closer to an insulating material than a semiconductor. At the opposite, GdCeO_2_S nanoplatelets feature a strong absorption threshold over 530 nm, corresponding to an estimated bandgap of 2.3 eV characteristic of semi-conductor. In summary, nanoparticles containing more Ce are expected to absorb a larger part of the visible light, which in turn could enhance their ability to produce ROS per se. 

### 2.2. ROS Production in Abiotic Conditions with Gd_2(1−x)_Ce_2x_O_2_S Nanoparticles Compared to BiVO_4_ Nanoparticles

Dithiothreitol (DTT) was used as molecular probe to evaluate production of reactive oxygen species (ROS) in the cellular media containing particles (see Section 5) [36,37,38]. The radical production was evaluated by calculating the difference between the normalized DTT quantities in the dark and under irradiation. For the radical production of Gd_2(1−*x*)_Ce_2*x*_O_2_S nanoparticles, we used nine LEDs (light-emitting diode) as light source (Figure S2). Their emission was centered at 501 nm with a FWHM (full width at half maximum) of ca. 15 nm. DTT self-photodegradation cannot occur under the conditions of this experiment since such molecules only absorb in the UV region. We first carried out the control test in the absence of nanoparticles. The absorbance values in the dark and under irradiation were comparable: 1.48 ± 0.01 and 1.44 ± 0.03, respectively. We confirmed that DTT is stable under irradiation with 501 nm light. Below, we display the value C/C_0_ on the graphs, which corresponds to the DTT concentration after incubation normalized vs. the DTT concentration before incubation.

For this study, five samples of nanoparticles were selected, with 0, 5, 10, 20 and 50% of Ce vs. Gd, as this allowed to cover a relevant broad range of bandgaps for visible light applications. Above 50%, the stability of the nanoparticles in air and in water-containing medium is not insured anymore [31], so we avoided this range of composition.

**Figure 3:**
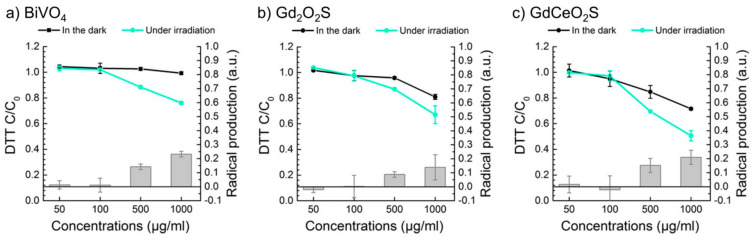
Radical production under irradiation of 501 nm visible light of (**a**) BiVO_4_ nanoparticles, (**b**) Gd_2_O_2_S (*x* = 0%) nanoparticles and (**c**) GdCeO_2_S (*x* = 50%) nanoparticles at different concentrations of photocatalysts. Radical production under irradiation, calculated as difference between the normalized DTT quantities in the dark and under irradiation, is presented as bar graph (right *y*-axis).

In order to evaluate the ROS production of Gd_2(1−*x*)_Ce_2*x*_O_2_S nanoparticles, a reference photocatalyst was required for comparison. Because TiO_2_ P25 nanoparticles are only active under UV light, we selected BiVO_4_ nanoparticles, previously synthesized in our lab, as a sample to be compared with our (Gd,Ce)_2_O_2_S nanoparticles [39]. The BiVO_4_ nanoparticles exhibit a small bandgap of 2.5 eV similar to those of GdCeO_2_S nanoparticles (2.3 eV) and could also be excited by 501 nm light. They generate radicals under irradiation and efficiently degrade rhodamine B via both photosensitization and photocatalysis. BiVO_4_ nanoparticles did not produce radicals in the dark (Figure 3a, black dots). In the dark, slight decreased of observed DTT is attributed to DTT adsorption on the nanoparticles. Under irradiation, less DTT was observed at higher concentrations of nanoparticles (500–1000 µg/mL) (Figure 3a, cyan dots). In particular, radical production of BiVO_4_ nanoparticles at 1000 µg/mL reached 0.2 (Figure 3a, grey bars).

The normalized DTT quantity in the dark with Gd_2_O_2_S (0% Ce) and GdCeO_2_S (50% Ce vs. Gd) nanoparticles is shown by black dots on Figure 3b,c, respectively. With increasing concentrations of nanoparticles, DTT quantity decreased to below 1 and DTT adsorption is stronger on GdCeO_2_S than on BiVO_4_ nanoparticles but slightly more than on Gd_2_O_2_S nanoparticles, which may be due to the higher specific surface of the oxysulfide and to a potential stronger interaction with the DTT molecule due to cerium introduction. The DTT adsorption difference between Gd_2_O_2_S and GdCeO_2_S in the dark is probably due to the difference of surface specific area (55 and 70 m^2^/g, respectively; see Supplementary Materials, Section S4).

Under irradiation, the DTT amount decreased significantly further (cyan dots). At the highest nanoparticle concentration (1000 µg/mL), the radical productions were 0.1 and 0.2 for Gd_2_O_2_S and GdCeO_2_S, respectively (grey bars). However, the fairly high dispersion of experimental values in the case of Gd_2_O_2_S nanoparticles (standard deviation of 0.15 at 1000 µg/mL) makes it difficult to confirm whether these can photogenerate radicals. In contrast, GdCeO_2_S nanoparticles produce radicals under irradiation with 501 nm light.

In order to better understand the role of cerium in radical production, the same experiment was carried out for Gd_2(1−*x*)_Ce_2*x*_O_2_S nanoparticles with intermediate cerium contents (1, 5, 10, 20%). The results using nanoparticles at 1000 µg/mL are summarized in Figure S3. Normalized DTT quantities varied from 0.4 to 1.0. For all samples containing cerium, the DTT amount was significantly lower under irradiation than in the dark, confirming the existence of a photocatalytic radical production. However, no clear trend related to the cerium content was observed. By contrast, Gd_2_O_2_S that absorbs mostly in the UV domain still showed a photogeneration of ROS under illumination.

To summarize, despite an effect of the aggregation state of the nanoparticles in the culture medium (likely related to their specific surface as pristine powders), visually observed in the culture medium, a careful comparison of results between cerium-containing and cerium-free nanoparticles, in the dark and under visible light, highlighted a significant positive effect of cerium on the formation of ROS by the nanoparticles. This was observed regardless of the Ce content, and no simple correlation between Ce content and the amount of ROS produced could be made at this stage. The presence of cerium was related to an increased photogeneration of radicals under 501 nm light, detected through the degradation of DTT in abiotic conditions. This result is consistent with that observed also for a reference BiVO_4_ photocatalyst, in which the radical generation is increased under irradiation [39]. By contrast, Gd_2_O_2_S that absorbs mostly in the UV domain showed a more moderate photogeneration of ROS under illumination, as the amount of ROS produced was significantly lower than those with Ce-containing nanoparticles.

In the next step, the consequence of this radical production by the nanoparticles in abiotic conditions was evaluated in vitro, as a part of the safer-by-design approach. We decided to focus on the samples with 0%, 10% and 50% Cerium.

### 2.3. Cytotoxicity and ROS Production In Vitro of Gd_2(1−x)_Ce_2x_O_2_S Nanoparticles

#### 2.3.1. Cell Viability under Medium to High Doses of Nanoparticles

Immortalized RAW 264.7 cells were used as an appropriate model of murine macrophages [40]. Following exposure to selected nanoparticles at different concentrations (1, 10, 50, 100 µg/mL) during 24 h, Hoechst and WST-1 assays were performed to characterize cellular DNA content and mitochondrial activity, respectively. For the following studies, the range in composition was restricted to fewer samples, namely, 0% (no Ce), 10% (doping regime) and 50% Ce vs. Gd, as there was no argument suggesting that intermediate compositions should behave differently in a significant way. As the nanomaterials we study are emerging and still far from the market, we cannot rely on real life exposure scenarios to address their cytotoxic effects. Therefore, in the following experiments, we stuck to the experimental conditions classically used in toxicity studies (hence the 1 to 100 mg/L and 15′–24 h time points).

##### DNA Quantification

**Figure 4:**
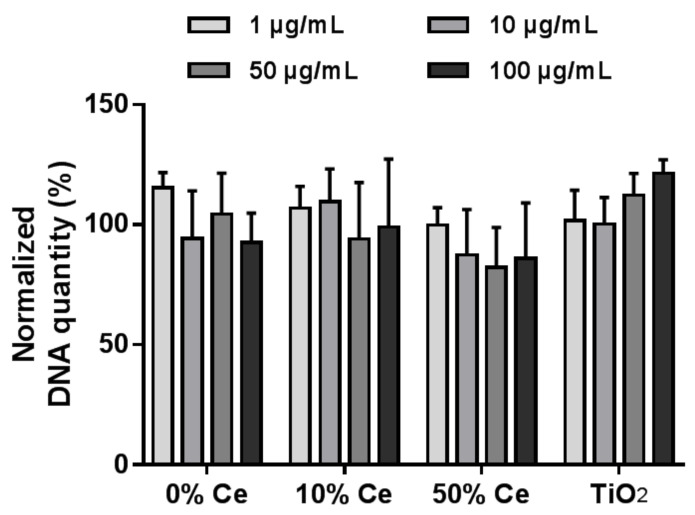
Cellular DNA quantification of RAW 264.7 macrophages exposed to Gd_2(1−*x*)_Ce*_x_*O_2_S and TiO_2_ P25 nanoparticles at different concentrations during 24 h. The cellular DNA contents were reported as percentages of that of unexposed cells. The experiments were repeated at least three times.

DNA quantification was performed after exposure of RAW 264.7 cells to Gd_2(1−*x*)_Ce_2*x*_O_2_S or TiO_2_ P25 nanoparticles at concentrations varying from 1 to 100 µg/mL (Figure 4). DNA quantification is classically used as a proxy for cell number, as it binds to double-stranded DNA. Interestingly, whatever the nanoparticles or the concentrations, no significant change in cellular DNA content was observed. The DNA contents remain at 100% (within the uncertainty interval) compared to unexposed cells.

##### Mitochondrial Activity

**Figure 5:**
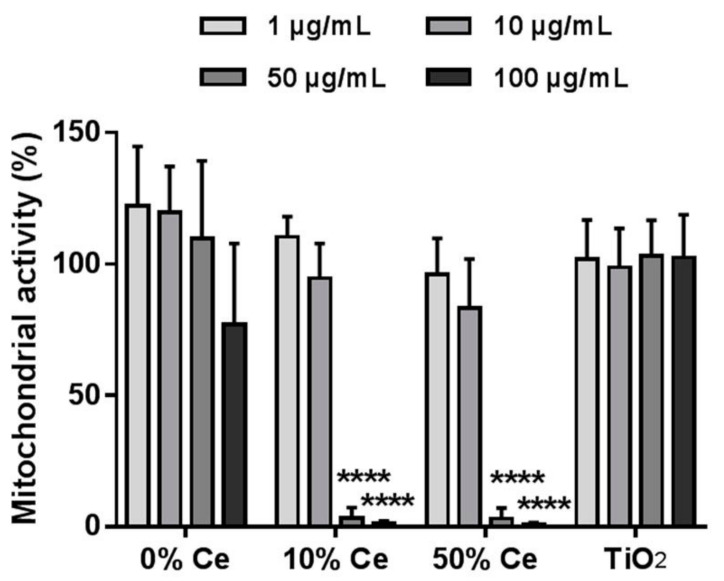
WST-1 assay of RAW 264.7 macrophages exposed to Gd_2(1−*x*)_Ce_2*x*_O_2_S and TiO_2_ P25 nanoparticles at different concentrations during 24 h. The mitochondrial activities were reported as percentages of that of unexposed cells. The experiments were repeated at least three times. **** signifies a *p*-value inferior to 0.0001.

The experiment was carried out for Gd_2(1−*x*)_Ce_2*x*_O_2_S nanoparticles with *x* = 0%, 10%, 50% of cerium as well as commercial TiO_2_ P25 nanoparticles as a control (Figure 5). The mitochondrial activity of RAW 264.7 macrophages was not affected upon exposure to TiO_2_ nanoparticles, whatever the concentration used. With Gd_2_O_2_S nanoparticles (0% Ce), mitochondrial activity was also preserved for concentrations of up to 50 µg/mL. At 100 µg/mL, a slight decrease in mitochondrial activity was observed, although not reaching statistical significance given the large uncertainty. For cerium-containing nanoparticles, the measured mitochondrial activity was unaffected by exposure to 1 or 10 µg/mL. However, exposure to higher concentration of nanoparticles (50 and 100 µg/mL) significantly decreases the mitochondrial activity of the cells down to 0% of that of unexposed cells.

#### 2.3.2. Sub-Lethal Effects of Nanoparticles In Vitro

We showed in the last section that severe damages to the cell were detected after exposure, for 24 h to 50 or 100 µg/mL of cerium-containing nanoparticles. However, at 10 µg/mL, these nanoparticles had no visible effect on the viability of the macrophages. Thus, we chose this latter concentration of nanoparticles as suitable for sub-lethal effect studies. Shorter duration of nanoparticles exposure (below 24 h) are also relevant to this purpose.

##### Intracellular ROS Production

**Figure 6:**
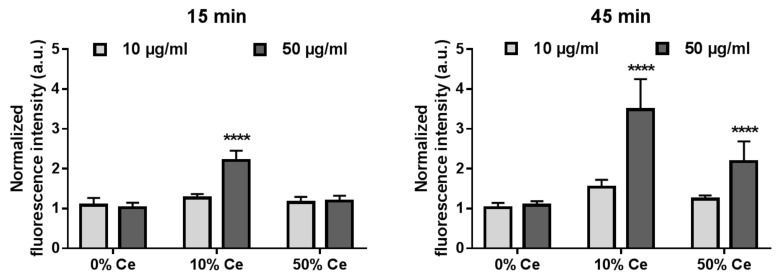
Intracellular ROS production of RAW 264.7 cells upon exposure to 10 and 50 µg/mL of Gd_2(1−*x*)_Ce*_x_*O_2_S nanoparticles (0, 10 and 50% Ce) after 15 min and 45 min. The ROS production was normalized to that of unexposed cells. The experiments were repeated three times. **** indicates a *p*-value inferior to 0.0001.

First, we investigated the intracellular production of reactive oxygen species (ROS) by RAW 264.7 macrophages in response to the exposure to oxysulfide nanoparticles. For this purpose, we employed the H_2_DCF-DA assay, based on fluorescence spectroscopy (the principle is described in ESI). The results are presented in Figure 6.

Following exposure to 10 µg/mL of Gd_2(1−*x*)_Ce_2*x*_O_2_S nanoparticles during 15 min, the normalized fluorescence intensity remained at ca. 1 for all tested nanoparticles (Figure 6, left panel, light gray bars). Exposure to 50 µg/mL of Gd_1.8_Ce_0.2_O_2_S (10% Ce) nanoparticles (Figure 6, left panel, dark grey) significantly modified the fluorescence intensity of DCF compared to that of unexposed cells. Cells exposed to Gd_2_O_2_S (0% Ce) and GdCeO_2_S (50% Ce) nanoparticles showed normalized fluorescence intensities around 1. In contrast, a significant increase to ca. 2 was observed for cells exposed to Gd_1.8_Ce_0.2_O_2_S (10% Ce) nanoparticles. The results of the H_2_DCF-DA assay suggest that, at 50 µg/mL, the 10% cerium-containing nanoparticles induced an increase in intracellular ROS production as soon as 15 min after the beginning of exposure.

After 45 min of exposure, for Gd_2_O_2_S (0% Ce) and GdCeO_2_S (50% Ce) nanoparticles, the intensity stayed at ca. 1. The fluorescence intensity was slightly but not significantly increased above 1 for nanoparticles with 10% Ce at 10 µg/mL. However, the normalized fluorescence intensity increased to above 3 following exposure of cells to Gd_1.8_Ce_0.2_O_2_S (10% Ce) nanoparticles and to above 2 in the case of GdCeO_2_S (50% Ce) nanoparticles at higher concentration after 45 min.

Overall, the observed effect of increased ROS production as a consequence of the presence of cerium cations in the compounds, observed here in abiotic conditions as well as in macrophages in vitro, stands in contrast with in vitro studies on ceria in the literature that suggest a suppressed ROS production [16], the ceria nanoparticles acting as superoxide dismutase mimics [30]. We speculated that oxidative stress could be one of the pathways that lead to death of RAW 264.7 macrophages upon exposure to cerium-containing Gd_2(1−*x*)_Ce*_x_*O_2_S nanoparticles. Hence, we investigated the expression of specific protein in response to oxidative stress.

##### Oxidative Stress

**Figure 7:**
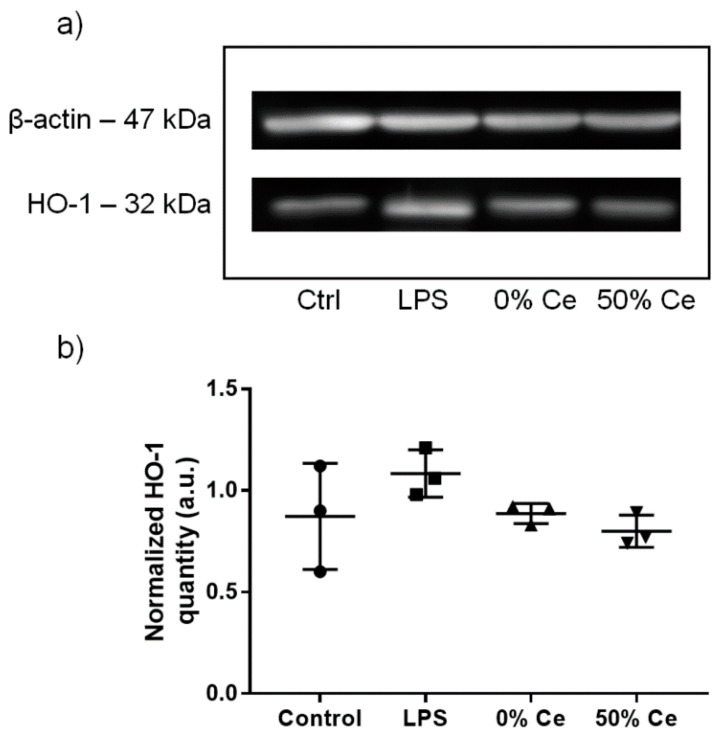
HO-1 expression of RAW 264.7 cells after 24 h of exposure to 10 µg/mL of Gd_2_O_2_S (0% Ce) nanoparticles and GdCeO_2_S (50% Ce) nanoparticles. Cell treatment with 10 µg/mL of LPS was used as positive control. (**a**) Results from western blot analysis of HO-1 and β-actin. (**b**) Average quantities of HO-1 deduced from the intensity of the fluorescent bands of three repeated western blot analyses. They are normalized with the corresponding β-actin quantity.

As for viability assays, we exposed RAW 264.7 macrophages to 10 µg/mL of Gd_2_O_2_S (0% Ce) nanoparticles or GdCeO_2_S (50% Ce) nanoparticles for 24 h. Western blot analysis showed the presence of HO-1 (heme oxygenase-1, a major antioxidant protein, PMID: 16129699) by chemiluminescence bands at ca. 32 kDa for untreated as well as treated cells (Figure 7a). Comparison between the chemiluminescence intensities of HO-1 band suggests a higher expression of HO-1 in the cells treated with LPS (lipopolysaccharide), used as positive control, than in untreated cells. Quantification of HO-1 expression (ratio to ß-actin expression, taken as housekeeping protein) of cells treated with nanoparticles were comparable to the control (Figure 7b).

##### Inflammatory Response

**Figure 8:**
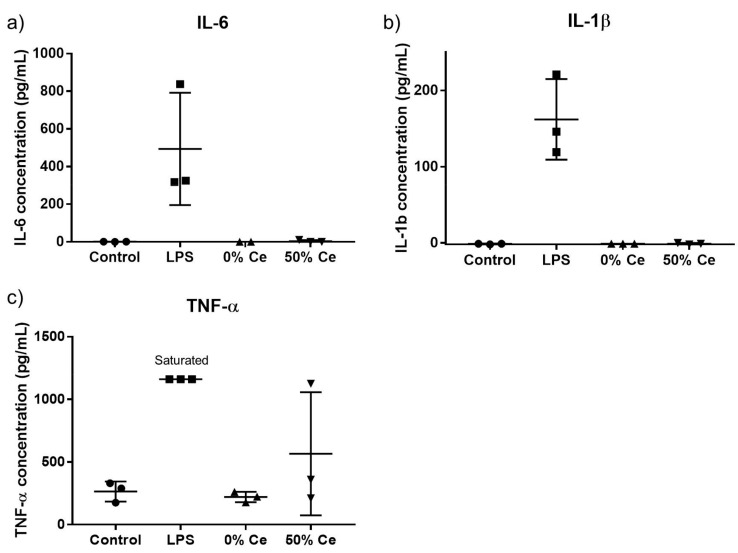
(**a**–**c**) Pro-inflammatory cytokines IL-6, IL-1β and TNF-α secretion levels of RAW 264.7 cells treated with 10 µg/mL of Gd_2_O_2_S (0% Ce) nanoparticles and GdCeO_2_S (50% Ce) nanoparticles during 24 h. Cell treatment with 10 µg/mL of LPS was used as positive control.

Besides oxidative stress, inflammation is also a relevant indicator of cellular response to nanoparticles exposure. In order to study the inflammatory response of the cell upon exposure to Gd_2(1−*x*)_Ce*_x_*O_2_S nanoparticles, we measured the expression of three pro-inflammatory cytokine. The results are presented in Figure 8.

The amount of IL-6 and IL-1β secreted by untreated cells were below the detection limit of the ELISA kit, although about 250 pg/mL of TNF-α was detected in the same sample. As expected, treatment with LPS significantly increased all three IL-6, IL-1β and TNF-α cytokine levels compared to untreated cells. However, the exact amount of secreted TNF-α could not be determined due to saturation of measured absorbance. Cells exposed to Gd_2_O_2_S (0% Ce) nanoparticles and GdCeO_2_S (50% Ce) showed no modification of IL-6 and IL-1β secretion, as for untreated cells. Compared to the control, the level of TNF-α was higher in cells exposed to GdCeO_2_S (50% Ce) nanoparticles while that in cell supernatant of cells exposed to Gd_2_O_2_S (0% Ce) nanoparticles was unchanged. The detected levels of inflammation markers IL-6, IL-1β and TNF-α in LPS-stimulated RAW 264.7 cells were comparable to those found in the literature, validating our experiment [41] However, given the high variability between the three independent experiments, we could not conclude on the exact statistical significance of this secretion.

### 2.4. Cerium Localization following the Incubation of Cells with Oxysulfide Nanoparticles

Given both the crystal structure and the hydrophobic envelope, any dissolution of the nanoparticles in the cell culture medium seemed unlikely. Moreover, any characterization of surface charge of the nanoparticles before/after the exposure of the cells could not be achieved due to the poor stability of dispersion and the aggregation state. In order to verify the particles integrity, co-localization of cerium and gadolinium was performed micro-X-ray fluorescence (µ-XRF) mapping of macrophage cells on the 50% Ce sample. The cells were incubated with GdCeO_2_S (50% Ce) nanoparticles at 10 µg/mL for 24 h at 37 °C.

**Figure 9:**
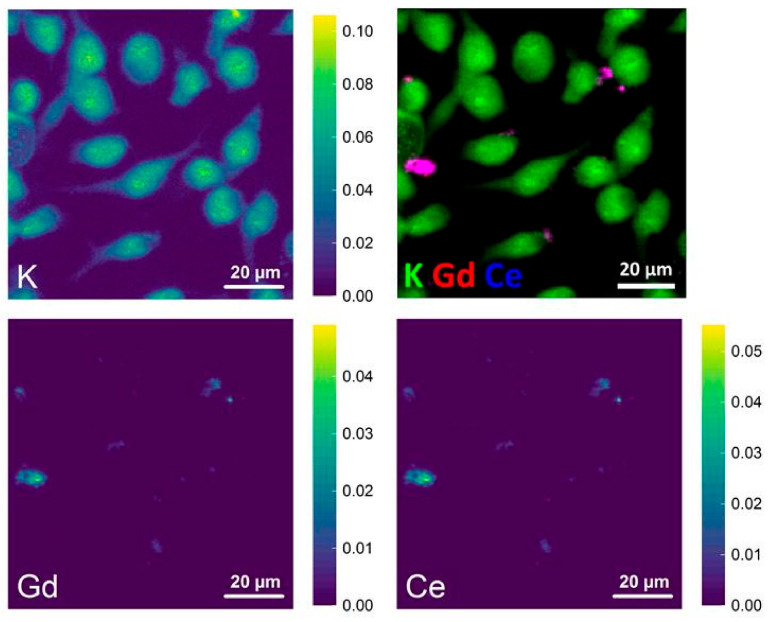
Single-element XRF maps (100 µm × 100 µm) of cells exposed to GdCeO_2_S (50% Ce) nanoparticles showing distribution of K (**top left**), Gd (**bottom left**) and Ce (**bottom right**). The images are displayed using a linear scale. The values in the color bars represent the concentration of elements in mM. The XRF maps were acquired at 7.4 keV. The merge color-coded map is also presented (**top right**).

Figure 9 shows single-element maps performed on this sample. The potassium signal is a good marker for localizing the biological material [42], as observed on the top left quadrant. The K map shows no change of morphology of the cell after 24 h of exposure to the nanoparticles (Figure 9 top left quadrant). Bottom quadrants show the two lanthanides. Because both elements are exogen to the cell (not present in the biological material), elemental signal of Ce and Gd come from nanoparticles or species degraded from the nanoparticles. Spots were both are detected in the expected 1:1 ratio should correspond to nanoparticles.

Regions of size ranging from 0.5 to 5 µm were observed with large amounts of nanoparticles. We attribute this as aggregates of nanoparticles, which are expected considering the hydrophobic layer around the nanoparticles. Larger aggregates were located farther from the cells. The color-coded merged map (top right quadrant) showed that both gadolinium and cerium were collocated in these regions. However, this depiction of the data did not allow discussing the regions containing lower amounts of Gd and Ce, where well-dispersed nanoparticles may be found.

**Figure 10:**
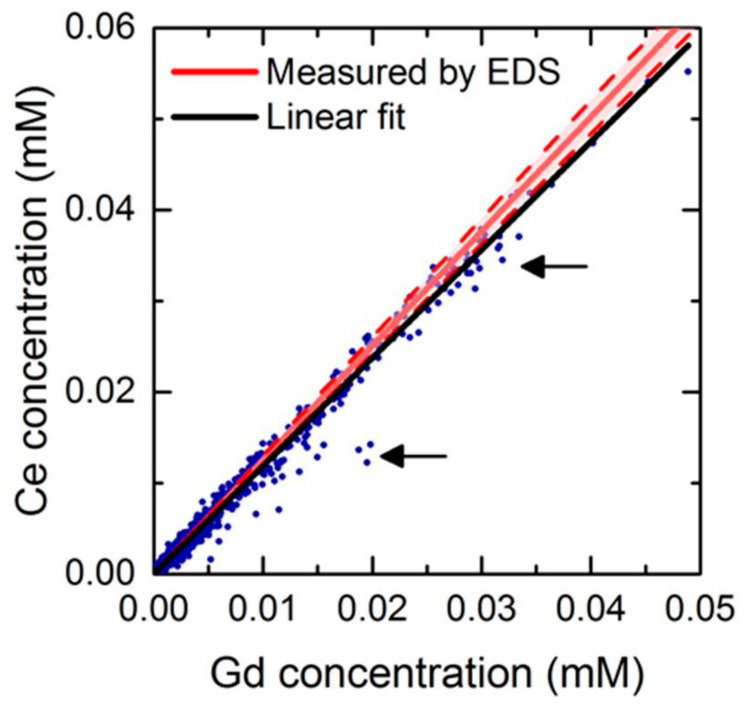
Scatter plot of concentration profile of Ce over Gd in XRF map of cells exposed to nanoparticles containing 50% of cerium. The red zones describe the standard deviation of the EDS measurement. The arrows indicate the groups of points that deviate from the linear concentration profile.

For this purpose, we plotted the concentration of Ce as function of the concentration of Gd at each pixel, in a so-called scatter plot (Figure 10). The majority of the points are aligned on the first bisector of the plot, confirming the co-localization between Gd and Ce not only for the large aggregates (top-right region of the scatter plot) but also for the diluted regions (bottom left region), that are observed in the larger number of pixels. Few dots are outside this linear profile, indicated with dark arrows.

In Figure 10, we also plotted the Ce/Gd ratio expected from the compositional analysis of the pristine nanoparticles powder (red line), measured by energy-dispersive X-ray spectroscopy (EDS): 1.25 ± 0.05. The linear fit from the scatter plot (black line) is close to this value, indicating that there is no major loss of cerium from the nanoparticles, regardless of their local concentration. This suggests a very low solubility of these nanoparticles in cellular media. Indeed, oxysulfides are considered more stable than their oxide counterparts due to the more covalent character of the M-S bond.

### 2.5. Impact of Cerium in ROS Production and Toxicity

In this work, our objective was to employ the safer-by-design approach on the gadolinium-cerium oxysulfide nanoparticles with particular attention given to cerium that is identified as cation regulating radical production and toxicity in other compounds such as ceria [30,42,43] and gadolinium-cerium oxides [28].

Nanoparticles interaction with cells is complex to interpret because of the multiple parameters to be considered: size, surface coverage, composition, crystalline structure, defects, etc. However, in the present study, the relevance of the results come from the fact that cerium-containing (Gd,Ce)_2_O_2_S nanoparticles are compared with Gd_2_O_2_S nanoparticles, all other parameters being as similar as possible from the synthesis point of view: average size, crystal structure, surface ligands (oleates), etc. The only predictable variable parameter is the partial substitution of Gd by Ce in the crystalline structure of the nanoparticles, in a known and controlled amount, a modification that may have affected other factors at the cellular level. In vitro studies were designed to evidence if this modification was correlated with a modification of any cytotoxicity parameter. First, with high doses of nanoparticles (up to 100 µg/mL), similar to these of the study by Dowding et al. on ceria [44], no loss of DNA was observed regardless of the cerium content or the dose. However, a significant loss of mitochondrial activity was evidenced, only using cerium-containing nanoparticles and at doses of 50 µg/mL or more, after 24 h of incubation. This allowed us to select a lower dose of 10 µg/mL as non cytotoxic for the following experiments.

Intracellular ROS activity was measured using shorter incubation times of 15 and 45 min. The presence of cerium was found to be a key factor, as cellular exposure to Gd_2_O_2_S did not result in a detectable ROS production, in contrast with three of the four other culture conditions. Although not obtained in the same experimental conditions (abiotic vs in vitro, concentration range, irradiation), this result is essential, as it connects abiotic and in vitro experiments: the presence of cerium is consistently associated with ROS production. The mechanisms at stake might be plural: charge generation as a consequence of light irradiation, redox activity of the Ce^III^/Ce^IV^ couple in the intracellular medium, etc. This result on gadolinium-cerium oxysulfides stands in contrast with in vitro studies on ceria (A549 cells) [30] and gadolinium-cerium oxide (Human foreskin fibroblasts AG01518) [28], the two closest materials available for comparison, which show an antioxidant effect of the nanoparticles.

From this milestone, two directions were explored. First, we investigated if the ROS production resulted in a detectable cellular response, either related to oxidative stress or to an inflammatory response. No significant effect was detected in our experimental conditions, whatever the endpoint. Second, we analyzed the question through the angle of the nanoparticles themselves, and their possible degradation in the culture medium. This was a challenging endeavor, as none of the regular characterization techniques used in material sciences (e.g., X-ray diffraction on powder) could be employed because of their small size and their high dilution. Preliminary results are, however, presented, based on µ-XRF that was performed on cryopreserved cells. This method of cell preservation is adequate to avoid the cells destruction, which is essential for the elemental cartography to be meaningful. This experiment confirmed the formation of region with higher concentration of nanoparticles (possibly, aggregates) in the cell culture, expected due to their hydrophobic character. Analysis by scatter plot also highlighted that the initial Ce/Gd ratio was preserved locally (at a submicronic scale), regardless of the local concentration of Gd and Ce and of their localization vs. the cells. We inferred from this result that no significant loss of cerium occurred as a result of incubation with cell. Consequently, we propose that the nanoparticle-cell interaction occurs from direct contact rather than from intermediate nanoparticle decomposition.

## 3. Implementation of Nano-Specific SbD Approach Using RATA

In the spirit of early SbD approach, we take risk analysis into account with the same emphasis as technological innovation at low TRL when the scientific research is beginning and before experimental proof of concept for a technology. This approach is more systemic than these usually found in typical studies on nanomaterials and is relevant to reduce the uncertainties and undesirable effects that may be caused by the circulation of new products that would employ emerging nanomaterials for their multi-functionality [17,26,27]. We have built this reflection as an example of a responsible innovation approach that can be integrated into a fundamental research project at the beginning of the value chain and life cycle. In this way, we are positioning the researcher as the first element in the chain of transmission of risk information related to safety and properties of new functional materials to the various stakeholders from designers to consumers.

Here, we propose a set of answers related to risk analysis and technological assessment (RATA), summarized in Table 1, for three Gd_2(1−*x*)_Ce_2*x*_O_2_S nanoparticles with *x* = 0%, 10%, 50% of cerium, deduced from the results of previous studies on characterization and properties of these materials and from the present work. Table 1 is constructed on an appropriate selection of the questions proposed by van Wezel et al. that apply more particularly to the RATA implementation of the SbD concept at the level of academic research. At this early stage of the assessment, we acknowledge that our study is to be considered with its limitations regarding the route of exposure: these routes were neither listed nor quantified in importance here, as this would have to be done in relation with the more applicative use of the nanoparticles.

First, we provide the “nano” characteristics of the materials we have synthesized (RA1) and then we recall the information given by the regulatory agencies of chemical substances related to their chemical composition only (RA2). All nanoparticles present nanosize with high aspect ratio by definition but their platelet morphology differentiates them from nanorods, nanotubes and nanowires that raise the same concerns as asbestos fibers and are considered as HARN (RA3) [45]. Therefore, we can say that these oxysulfides nanoparticles are not HARN.

The nomenclature on nanoform proposed by REACH according to the modified Annex VI of REACH (RA4) is more precise. It allows distinguishing particles with a high aspect ratio in several groups. Due to their high shape anisotropy, the three compounds enter the category of 2D nanomaterials and not of high aspect ratio nanomaterials (RA4). Only Gd_2_O_2_S had a CAS number and was already known by the European chemicals agency (InfoCard 100.032-350) that states that this substance causes serious eye and skin irritation, is harmful if swallowed, inhaled or in contact with skin, and may also cause respiratory irritation. Cerium doped Gd_2_O_2_S compounds are new and not yet listed by regulatory agencies (RA2).

By the following, we report new information related to this study that increases our level of knowledge in the risk analysis of similar products already listed (RA4 to RA9). More specifically, the solubility is compared to already known similar oxide materials in terms of energy bonding. Indeed, the M-S bonding in metal sulfides has a more covalent character than M-O in metal oxide suggesting that metal oxysulfides are more covalent than similar metal oxides and less soluble. Information on the environmental fate and behavior of nanoparticles is given with consideration to their very small size, their solubility and their capacity to generate ROS under visible light in the water compartment. Here, we take into account the toxicity that could be induced by the release of gadolinium and cerium ions, and the oxidative stress that could be induced by the accumulation of these particles on microorganisms and plants in the different compartments, respectively (RA8–9).

This RATA approach shows all the interest of toxicity studies very early in the innovation process and allows better classification of emerging materials. For the materials of the present study, a dichotomy in the field of potential applications and in the evaluation of the hazard through the control of risk and exposure stands out very well according to whether or not the material contains cerium. Nevertheless, if the approach can be fruitful, its application to emerging nanomaterials, freshly out of the laboratory and not yet on the market, represents a shift in paradigm that it is important to initiate also in fundamental research. The long-term motivation concerns the trust that society, economic and political actors, may place in further technological developments.

## 4. Conclusions

In nanotechnology, the safer-by-design approach attempts at performing alongside the development of the nanoparticle’s qualities, varying for instance their composition or their surface coverage, and the assessment of each of these parameters on their safe use. We employed this process on an emerging family of materials, the lanthanide oxysulfides, scarcely studied and not yet manufactured. Here, we focused on one quality: the bandgap, which is tuned by the addition of cerium in the compound and is critical to a range of future applications such as photocatalysis or in the fields of semi-conductors. We selected one key parameter, tunable by design: the amount of cerium substituted to the gadolinium in the inorganic structure of the oxysulfide.

Our results show that the impact of cerium differs from that of reported systems such as ceria and gadolinium-cerium oxides. In particular, the production of ROS is increased by the incorporation of cerium, even with sub-lethal dose of nanoparticles. So far, our study did not highlight any cerium dissolution in the cell culture, although this may have to be assessed with more sensitive techniques in the future.

All the results of this study are then integrated through a risk analysis and technology assessment (RATA). The aim is to ensure that academic research is more responsible in order to make the implementation of new technologies more acceptable as part of an ethical and civic approach. These recent initiatives reflect the awareness of research workers of their capacity to reform practices and to build a responsible innovation and research approach relating to the emergence of new technologies that could be integrated in future research programs.

## 5. Experimental Section

### 5.1. Nanoparticles Synthesis

Nanoparticle’s synthesis was performed according to a published procedure.^31^ Oleylamine (OAm; technical grade, 70%), oleic acid (OA; technical grade, 90%), 1-octadecene (ODE; technical grade, 90%), sulfur (S_8_; ≥99.5%), sodium oleate (Na(oleate); ≥99%), were purchased from Sigma-Aldrich. Gadolinium(III) acetylacetonate (Gd(acac)_3_∙*x*H_2_O; 99.9%) and cerium(III) acetylacetonate (Ce(acac)_3_∙*x*H_2_O; 99.9%) were purchased from Strem Chemicals. The latter was stored in the glovebox. All chemicals described above were used without further purification.

Oxysulfide nanoparticles were prepared via a solvothermal reaction under purified nitrogen atmosphere using standard air-free techniques with Schlenk line. In a typical synthesis using sodium acetylacetonate, Gd(acac)_3_∙*x*H_2_O (227 mg, 0.5 mmol, 1 equiv.), S_8_ (8 mg, 0.032 mmol, 0.5 equiv. in S), Na(oleate)∙*x*H_2_O (76 mg, 0.25 mmol, 0.5 equiv.) were added to a mixture of OA (0.71 g, 2.5 mmol, 5 equiv.), Oam (4.54 g, 17 mmol, 34 equiv.) and ODE (8.10 g, 32.5 mmol, 65 equiv.) in a 100 mL three-necked flask at room temperature. The yellow mixture was degassed at 120 °C under vacuum for 20 min then heated to 310 °C, giving a pale-yellow solution. The solution was stirred at this temperature for 30 min under nitrogen. After heating, it was left to cool to room temperature.

The nanoparticles were Isolated by centrifugation (6000× *g*, 10 min, 20 °C) using 30 mL of ethanol. They were washed at least three times using 40 mL of a *n*-hexane/ethanol mixture (1/3 in volume) to remove remaining reagents and organic matter. From 100 to 120 mg of dried white powder were obtained, corresponding to a 100% yield of Gd_2_O_2_S (this calculation neglects the weight of organic ligands).

Synthesis of (Gd,Ce)_2_O_2_S nanoparticles was carried out in the same fashion. Added quantities of gadolinium precursor Gd(acac)_3_∙*x*H_2_O, cerium precursor Ce(acac)_3_∙*x*H_2_O were adjusted accordingly so that the total amount of lanthanides added was 0.5 mmol. For these syntheses, from 50 mg to 100 mg of dried powder were obtained depending on the cerium content of the nanoparticles.

### 5.2. UV-Visible Spectroscopy

Measurements in liquid mode were carried out using suspensions of nanoparticles loaded in a 3.5 mL absorption quartz cell with an optical path length of 10 mm. Absorption spectra were recorded using a Cary-WinUV 5000 spectrophotometer (AGILENT) between 300 nm and 800 nm with steps of 1 nm. For measurements in diffuse reflectance mode, dry powders of nanoparticles were loaded in the sample holder to make a uniform layer of solid. The UV-visible diffuse reflectance spectra were measured using an integration sphere between 250 nm and 800 nm at 1 nm.s^−1^ and corrected with a sample of BaSO_4_ as reference. The Kubelka-Munk function was calculated from obtained apparent absorbance according to the formula:

$$F\left(R\right)=\frac{{\left(1-R\right)}^{2}}{2R}$$

where *R* is the reflectance. The apparent absorbance given by the spectrometer is related to the reflectance by the following equation:

A=log1R

### 5.3. Nanoparticle Dispersion for Cellular Studies

Nanoparticle stock suspensions (5 mg/mL) were prepared by dispersing 25 mg of dry powder of nanoparticles in 5 mL of sterile water with the help of sonication. The stock suspension was then divided into aliquots of 1 mL, and they were stored at 4 °C. Prior to conducting cellular studies, the suspensions were sonicated for 30 min.

### 5.4. Detection of ROS Using DTT

In the presence of ROS, DTT transforms into its radical form DTT-S^•^ which dimerizes into the corresponding disulfide (ox-DTT). The remaining DTT is reacted with 5,5′-dithiobis-(2-nitrobenzoic acid) (DTNB) and the colored product of this reaction, the 2-nitro-5-thiobenzoic acid (TNB, λ_max_ = 405 nm), is quantified by UV-visible absorption spectroscopy. A high number of produced radicals results in a small quantity of TNB detected. The principle of the test is detailed in the Supporting Information.

We adapted an experimental protocol for 96-well plate to screen the photocatalytic activities of (Gd,Ce)_2_O_2_S nanoparticles. Aqueous DTT solution and suspension of nanoparticles in water were added in each well of a 96-well plate in duplicate. One plate was irradiated by light from LEDs during 4 h while the other was left in the dark during the same amount of time. The (Gd,Ce)_2_O_2_S nanoparticles containing cerium absorb at the wavelength used to detect TNB (405 nm). Thus, the plates were centrifuged to sediment the nanoparticles and only the supernatant was taken and transferred to new plates. Excess DTNB is then added to the supernatant to form TNB in a quantitative reaction. The absorbance at 405 nm was finally measured for each well.

### 5.5. Cell Culture

Murine macrophages RAW 264.7 (ATCC) were maintained in Dulbecco’s modified Eagle’s medium (DMEM) containing 4.5 g/L of glucose, 10% fetal bovine serum (FBS), 100 U/mL penicillin and 100 U/mL streptomycin at 37 °C, 5% CO_2_ atmosphere. The cells were cultured in 75 cm^2^ flask for 2 or 3 days in the dark and harvested by scraping. They were then cultured in 96-well plates for cell viability assays or onto silicon nitride membrane for X-ray hyperspectral imaging.

### 5.6. Cell Viability Assays

#### 5.6.1. General Considerations

For viability assays, 15,000 cells were seeded in a 96-well plate. The nanoparticle stock suspensions were diluted in DMEM medium without phenol red to avoid interference of the latter with subsequent colorimetric and fluorescent assays. A series of suspensions at different concentrations (1, 10, 50, 100 µg/mL) was prepared. After 24 h of culture at 37 °C, the cells were washed with fresh DMEM medium without phenol red and 100 µL of the previously prepared nanoparticle suspensions were added. Cells exposed to nanoparticles were then incubated for another 24 h. Mitochondrial activity, cellular DNA content and membrane integrity were respectively assessed by WST-1 (Roche), Hoechst (Sigma-Aldrich) and LDH (Roche) assays using a TECAN microplate spectrometer.

For WST-1 assay, which indicates cell viability, the cell culture medium was removed at the end of nanoparticle exposure and the plate was washed twice with fresh medium. Then, 100 µL of the WST-1 solution (concentration not provided by the manufacturer) were added to each well. After 3 h of incubation at 37 °C, the absorbance of the medium was measured at 450 nm.

For Hoechst assay, the cell culture medium was removed at the end of nanoparticle exposure and the plate was washed twice with Dulbecco’s phosphate-buffered saline (DPBS) with no calcium or magnesium. Then, the cells were incubated with 20 µL of sterile water for 45 min at 37 °C. At that time, 200 µL of Hoechst 33,258 solution (2 µg/mL) in fluorescence buffer (provided by the manufacturer) was added. The fluorescence intensity of the medium was measured at 460 nm with excitation at 360 nm.

#### 5.6.2. DNA Quantification

DNA quantification was performed by fluorometry using the Hoechst 33258 dye (see Supplementary Materials, Section S7). RAW 264.7 macrophages, which were exposed to nanoparticles in microplates, were incubated with water for 30 min. This cytolysis step was necessary to release intracellular DNA in the culture medium. Then, a solution of Hoechst dye was added, and fluorescence intensity was measured in each well with a microplate reader. The assay was performed with Gd_2(1−*x*)_Ce_2*x*_O_2_S and TiO_2_ P25 nanoparticles at different concentrations from 1 to 100 µg/mL (Figure 4). The fluorescence intensity was normalized to that of unexposed cells.

#### 5.6.3. Mitochondrial Activity

In this experiment, RAW 264.7 cells were exposed to nanoparticles for 24 h. The exposed-cells were incubated with WST-1 solution for 3 h (the principle of the WST-1 assay is described in Supplementary Materials, Section S6). The absorption of resulting media was measured by a microplate reader at 450 nm and the relative mitochondrial activity was calculated by normalizing the absorbance values to that of unexposed cells.

### 5.7. Intracellular ROS Activity Assessment

Assessment of intracellular ROS activity was carried out using H_2_DCF-DA assay (Molecular Probes by ThermoFisher). In a similar fashion to cell viability assays, 15,000 cells were seeded in a 96-well plate for 24 h. They were washed twice with Hank’s Balanced Salt Solution (HBSS) with no calcium nor magnesium. The cells were then incubated with 100 µL of a H_2_DCF-DA solution at 10 µM for 1 h at 37 °C. After the incubation, the supernatant was removed, and the plate was washed twice with fresh HBSS. The cells were treated with 100 µL of Gd_2_O_2_S and Gd_2(1−*x*)_Ce_2*x*_O_2_S nanoparticles (10 and 50% of cerium) suspensions at different concentrations. The fluorescence intensity of the medium was measured at 530 nm before nanoparticle treatment and at 15 min and 45 min after the treatment. The excitation light was fixed at 485 nm. The measured fluorescence intensities were normalized to that of unexposed cells.

### 5.8. Protein Expression Analysis

In a 6-well plates, 10^6^ cells were seeded and cultured for 24 h. After the incubation, the cells were washed with fresh medium without phenol red and 2 mL of nanoparticle suspension in DMEM medium without phenol red was added. After 24 h of treatment with 10 µg/mL of Gd_2_O_2_S (0% Ce) nanoparticles or GdCeO_2_S (50% Ce) nanoparticles, the cells were collected by scraping with RIPA mammalian protein extraction lysis buffer (Sigma-Aldrich) with 10% of protease inhibitor cocktail (Sigma-Aldrich) and 10% of phosphatase inhibitor cocktails (Sigma-Aldrich) to prevent protein degradation and dephosphorylation by endogenous proteases and phosphatases present in the whole cell extract. The obtained mixtures were centrifuged to remove cell debris and nanoparticles. The supernatant was stored at −80 °C for protein analysis. The total protein concentration in the supernatant was determined by Bradford assay (Bio-Rad) with bovine serum albumin (BSA) (Sigma-Aldrich) as the standard.

The analysis of protein expression was carried out by western blotting. For this experiment, the HO-1 antibody (rabbit) was purchased from Enzo Life Sciences (concentration not provided by the manufacturer) and was diluted 1000 times in a solution of BSA 1%, while the β-actin antibody (mouse) was purchased from Sigma-Aldrich and was diluted 5000 times in a solution of milk 5%. In a typical experiment, 40 µL of a solution containing 30 µg of protein in Laemmli buffer was loaded on a sodium dodecyl sulfate-polyacrylamide gel electrophoresis (SDS-PAGE) gel (4% and 10% of acrylamide for stacking and resolving parts of the gel, respectively). The gel was run at a constant voltage of 60 V for 1 h then at 120 V for 1.5 h. The proteins were transferred to a nitrocellulose membrane by electroblotting at 20 V, 4 °C for 18 h. Blocking of unreacted sites of the membrane was performed by incubation with a solution of milk 5% for 1 h. The membrane was incubated with primary antibody (HO-1 or β-actin) at 4 °C for 16 h then with secondary antibody coupled with peroxidase ECL or alkaline phosphatase at room temperature for 2 h. It was finally reacted in the dark with peroxidase substrate (ECL, Bio-Rad) or alkaline phosphatase (Bio-Rad) for revelation of HO-1 or β-actin, respectively. Detection of luminescence signals and acquisition of images of the membrane were performed on a G-box (Syngene, UK). Quantification of luminescence intensity was carried out with ImageJ software.

As a positive control, the cells were treated with 10 µg/mL of lipopolysaccharide (LPS), an endotoxin. The whole experiment was repeated twice, and no significant difference was observed.

### 5.9. Measurement of Pro-Inflammatory Cytokine Secretion

In order to study the inflammatory response of the cell upon exposure to Gd_2(1−*x*)_Ce*_x_*O_2_S nanoparticles, we measured the pro-inflammatory cytokine levels. In particular, interleukin 6 (IL-6), interleukin 1beta (IL-1β) and tumor necrosis factor alpha (TNF-α) are important inflammation markers and were chosen to be studied in a preliminary study.

In a 6-well plates, 10^6^ cells were seeded and cultured for 24 h. After the incubation with the nanoparticles at 10 µg/mL during 24 h, the cells were washed with fresh medium without phenol red and 2 mL of nanoparticle suspension in DMEM medium without phenol red was added. After 24 h of treatment, the supernatant was taken and stored at −80 °C for assessing cytokine and chemokine levels.

The IL-6, IL-1β and TNF-α pro-inflammatory cytokines was quantified using the supernatant collected from the treated cells. LPS was employed as positive control. The analyses were conducted using Quantikine ELISA kits purchased from R&D Systems. The measurement of the secreted cytokine level involved transferring 50 µL of the collected cell culture supernatant into 96-well plates coated with the capture antibody against the targeted cytokine. Then a detection antibody was added and bound to the captured cytokine. Unbound detection antibody was washed away. The sandwich capture antibody-cytokine-detection antibody was revealed by adding a tetramethylbenzidine (TMB) substrate solution to develop a blue color. A hydrochloric acid solution provided by the manufacturer was added to stop the reaction and the color turned yellow. The detailed protocol was based on the manual provided in the kit purchased. The cytokine levels were quantified by measuring absorbance of the medium at 450 nm using a TECAN microplate spectrometer.

### 5.10. X-ray Fluorescence (XRF) Imaging

This analysis was performed on beamline ID21 at ESRF in Grenoble. A sample incubated with nanoparticles containing 50% Ce and then cryopreserved was analyzed, as well as the control sample.

Murine macrophages RAW 264.7 were seeded at concentration of 4.10^5^ cells/mL onto 200 nm thick silicon nitride (Si_3_N_4_) membrane windows (Silson Ltd., Southam, UK) placed in 12-well plates. After 24 h of culture at 37 °C, they were washed with fresh medium and were exposed to suspensions of 10 µg/mL of Gd_2(1−*x*)_Ce*_x_*O_2_S (0, 10 and 50% Ce) nanoparticles, similar to the previous sub-lethal effect studies. RAW 264.7 cells similarly grown onto the windows, but not exposed to nanoparticles (untreated cells), were used as control (see Figure S9).

After another 24 h of incubation, the membranes were washed with Dulbecco’s phosphate-buffered saline (DPBS) with no calcium or magnesium, then rapidly with water. The water was removed by blotting on a Kimwipes paper without direct contact to the thin membrane. The windows were then snap-frozen by plunging into isopentane cooled by liquid nitrogen for 30 s. They were stored in 24-well plates cooled at −80 °C in a freezer or with dry ice.

X-ray hyperspectral imaging experiments were carried out at ID21 beamline of European Synchrotron Radiation Facility (ESRF) synchrotron (Grenoble, France) with the help of Dr. Ana Elena Pradas del Real, Dr. Hiram Castillo and Dr. Murielle Salomé. The beamline is equipped with a vacuum chamber passively cooled at 130 K by liquid nitrogen. The X-ray beam was tuned to 7.4 keV with a Si(111) two-crystal monochromator. Emitted X ray Fluorescence was recorded by a silicon drift detector (SGX Sensortec 80 mm^2^ active area) and a Si_3_N_7_ diode was used to record I_0_ signal. The silicon nitride (Si_3_N_4_) membrane windows were mounted onto a pre-cooled copper sample holder immersed in liquid nitrogen and were rapidly inserted into the vacuum chamber. µXRF maps were acquired with 0.5 µm^2^ steps and an integration time of 100–150 ms.

Analysis of the XRF images and spectra was carried out using the multiplatform program PyMCA [46]. Elemental mass fractions were calculated from fundamental parameters with the PyMca software package, applying pixel-by-pixel spectral deconvolution to hyperspectral maps normalized by the incoming current. The detector response was calibrated using reference sample on Si_3_N_4_ membrane RF8-200-52454-17 purchased from AXO DRESDEN GmbH (Dresden, Germany). To calculate weight fractions, the thickness of the cells and the density of the medium were estimated at 15 µm and 1 g/mL, respectively.

## Figures and Tables

**Table 1:** Risk Analysis and Technology Assessment of Gd_2(1−*x*)_Ce_2*x*_O_2_S nanoparticles inspired from [27] by applying van Wezel methodology [26] for SbD considerations.

	Case Study	Gd_2_O_2_S	Gd_1.8_Ce_0.2_O_2_S	GdCeO_2_S
**Risk analysis**	RA1. What is the «nano» aspect of your development?	Crystalline nanoplatelets (2D nanomaterial) [31] Size: width 20 ± 5 nm/thickness 2 ± 1 nm [31] Surface state: coordination of oleate ligands [34]		
	RA2. What is the already known regulatory framework?	CAS: 12339-07-0 ECHA info card: 100.032-350 (eye, lung and skin irritant, harmful if swallowed or inhaled).	CAS: not yet No harmonized classification yet	CAS: not yet No harmonized classification yet
	RA3. What do you already know on the safety aspects?	HARN *: No No cytotoxicity on Murine macrophages RAW 264.7 (up to 100 µg/mL after 24 h) **. No inflammatory response at 10 µg/mL after 24 h **.	HARN *: No Cytotoxic on Murine macrophages RAW 264.7 (>10 µg/mL after 24 h) **. Induced strong oxidative stress at 50 µg/L after 15 min **.	HARN *: No Cytotoxic on Murine macrophages RAW 264.7 (>10 µg/mL after 24 h) ** Induced oxidative stress at 50 µg/L after 15 min ** No inflammatory response at 10 µg/mL after 24 h **.
	RA4. Are there any discussion on “nano” within legislative framework?	REACH: 2D nanoform (hazard data between nanoforms and/or sets of nanoforms, and the non-nanoforms of the same substance) ***		
	RA5. What are new aspects, related to already authorized products?	Smaller size suggesting better biodistribution of contrast agent and clearance **	Antioxidant properties that could be adjusted by tuning Ce content **	Very small semiconductor with strong absorption band in visible **
	RA6. Is your product less risky than existing products regarding solubility?	Less soluble than Gd_2_O_3_		Less soluble than CeO_2_
	RA7. Do you have any information on the intrinsic hazardous aspects?	Strong adsorption capability due to surface reactivity and high surface area **		
		No lethal toxicity up to 100 µg/mL ** No intracellular ROS activity up to 50 µg/mL **	Lethal toxicity from 50 µg/mL ** Induced strong intracellular ROS activity above 10 µg/mL **	
	RA8. Can material be released in significant quantities during the production, use, or waste phase?	Low dissolution rate and Gd^3+^ release in water, biologic media and cell compartment at short term but not know at long term **		
	RA9. Do you have information on the environment fate and behavior?	Exposure: consider the very small size of nanoparticles in the exposure scenarios specific to the manufactured products and applications that will use these materials.		
		Low ROS production under visible light (501 nm) in water until 1000 µg/mL **	High ROS production under visible light (501 nm) in water over 100 µg/mL **	
**Technology assessment**	TA1. Which other stakeholders, besides suppliers and customers, could you imagine?	Pharmaceutical laboratory Medical doctors performing imaging for diagnosis	Pharmaceutical laboratory Medical doctors performing imaging for diagnosis and therapy	Depollution industry Alternative energy producers
	TA2. How will these stakeholders be affected in both positive and negative ways?	Insulator material	Semiconductor	Semiconductor
	TA3. How does this new technology influence stakeholder’s responsibilities and liabilities?	Potential use for biomedical application with controlled exposure	High reactivity suggesting restricted use for biomedical application—Requires effective surface protection	Application limiting exposure and contact with skin and eyes
	TA4. Which different possible futures could you imagine with your development?	Biomedical imaging: MRI contrast agent for diagnosis X-ray absorbing agent	Theranostics: mixing antoxidant properties of Ce with magnetic properties of Gd for MRI	Photocatalysis in visible light Electrolyte based materials

* High aspect ratio nanoparticles (HARN), the value is given in brackets. ** affirmation deduced from the present study. *** REACH: Appendix R.6-1 for nanoforms applicable to the Guidance on QSARs and Grouping of Chemicals.

## Data Availability

Not applicable.

## Supplementary Materials

The following are available online at https://www.mdpi.com/article/10.3390/nano12030422/s1, Figure S1: Reaction scheme of (1) DTT with DTNB to form an intense yellow product, detectable by UV-visible absorption spectroscopy and (2) DTT with radicals in solution to form disulfide ox-DTT that does not react with DTNB., Figure S2: Experimental setup, Figure S3: Normalized DTT quantities measured after 4 h in the dark and under irradiation with 1000 μg/mL of Gd_2(1-*x*)_Ce_2*x*_O_2_S nanoparticles (*x* from 0 to 50%), Table S1: Sodium contents measured by SEM-EDS and specific surface areas measured by adsorption isotherm of Gd_2(1-*x*)_Ce_2*x*_O_2_S nanoparticles with 0, 10 and 50% of cerium. The surface area values were extracted using the Brunauer-Emmett-Teller (BET) theory, Figure S4: Optical microscope image of murine macrophage RAW 264.7 cell line provided by American Type Culture Collection (ATCC^®^ TIB-71™), Figure S5: Cleavage of tetrazolium salt WST-1 to formazan by mitochondrial dehydrogenase. EC stands for electron coupling reagent, Figure S6: Assay interference test: incubation of formazan dye with 100 μg/mL of Gd_2(1-*x*)_Ce_2*x*_O_2_S nanoparticles. Resulting absorbance is normalized by that of formazan dye, Figure S7: Chemical formula of Hoechst 33258 molecule, Figure S8: Measuring intracellular ROS activity by H2DCF-DA assay, Figure S9: Single-element XRF maps of non-exposed cells showing distribution of K, P, S and Ca. The images are displayed using a linear scale. The values in the color bars represent the concentration of elements in mM. The XRF maps were acquired at 7.4 keV.
