# Supplementary material for: Risk Analysis and Technology Assessment of Emerging (Gd,Ce)2O2S Multifunctional Nanoparticles: An Attempt for Early Safer-by-Design Approach
Source: Nanomaterials (Basel). 2022 Jan 27;12(3):422. doi: 10.3390/nano12030422 (PMC8840297; doi:10.3390/nano12030422)
Supplement: Supplementary file 1 [file nanomaterials-12-00422-s001.zip › nanomaterials-1529652-supplementary.pdf]

nanomaterials

MDPI

Supplementary Materials

Risk Analysis and Technology Assessment of Emerging $(Gd,Ce)_2O_2S$ Multifunctional Nanoparticles: An Attempt for Early Safer-by-Design Approach

Anh-Minh Nguyen [1,2], Ana Elena Pradas del Real [3], Olivier Durupthy [1], Sophie Lanone [2,*], Corinne Chanéac [1,*] and Sophie Carenco [1,*]

[1] Laboratoire de Chimie de la Matière Condensée de Paris, Collège de France, Sorbonne Université, CNRS, 4 Place Jussieu, 75252 Paris, France; anhminh.nguyen@nexdot.fr (A.-M.N.); olivier.durupthy@sorbonne-universite.fr (O.D.)
[2] INSERM, IMRB, Univ Paris Est Creteil, , F-94010 Creteil, France
[3] ESRF, 71 Avenue des Martyrs, 38000 Grenoble, France; ana.elena.pradas@madrid.org (A.E.P.)
* Correspondence: sophie.lanone@inserm.fr (S.L.), corinne.chaneac@sorbonne-universite.fr (C.C.), sophie.carenco@sorbonne-universite.fr (S.C.)

1. Principle of the DTT Test

Detection of reactive oxygen species (ROS) such as hydroxyl radical •OH and superoxide radical $O_2^{\bullet-}$ under photocatalytic conditions has been carried out by a wide range of methods including spectroscopy, colorimetry, fluorescence, etc.[1] Standing out is EPR spin trapping method. It consists in trapping the radicals which have short lifetime in solution with molecules such as 5,5-dimethyl-1pyrroline *N*-oxide (DMPO). The formed adduct DMPO-R• has a longer lifetime and can be detected by EPR spectroscopy. This method was developed for selective and efficient detection of photogenerated radicals even at low concentrations.[2] Nevertheless, EPR spectroscopy requires specific and expensive equipment. Hence, alternative methods are also developed. Colorimetry and fluorescence using selective radical probes are the most commonly used. For example, tetrazolium (XTT) (colorimetry) is used for detection of $O_2^{\bullet-}$ while terephthalic acid (fluorescence) and p-nitrosodimethylaniline (RNO) (colorimetry) are used for •OH.[1]

In this work, dithiothreitol (DTT) molecules are used as radical probe. DTT can react with 5,5'-dithiobis-(2-nitrobenzoic acid) (DTNB or Ellman's reagent), cleaving the disulfide bond to form 2-nitro-5-thiobenzoic acid (TNB). This reaction between the two colorless compounds results in a product with intense yellow color (λ_{max} = 405 nm) (Figure S1-1). Thus, the quantity of present DTT can be measured using UV-visible absorption spectroscopy high sensitivity.

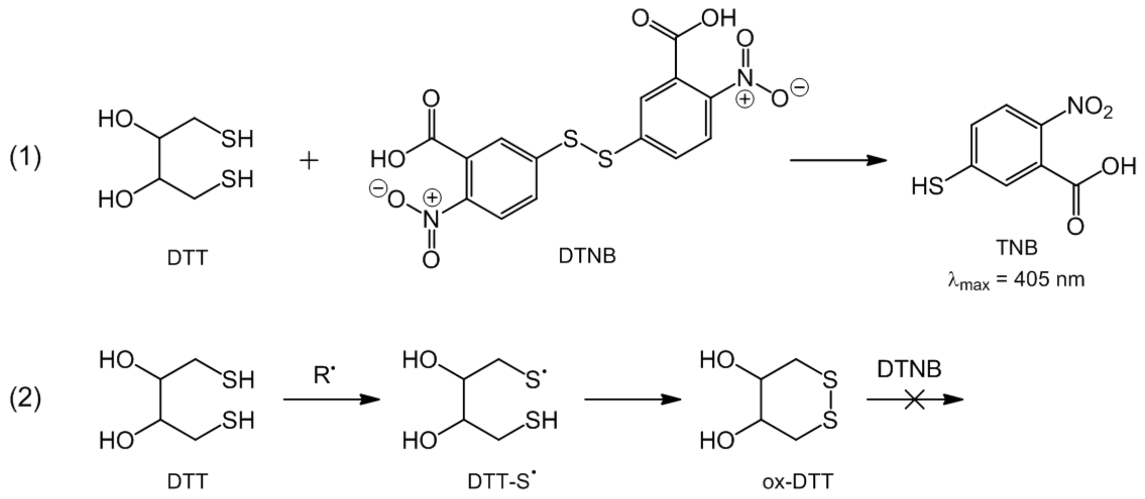

Figure S1. Reaction scheme of (**1**) DTT with DTNB to form an intense yellow product, detectable by UV-visible absorption spectroscopy and (**2**) DTT with radicals in solution to form disulfide ox-DTT that does not react with DTNB.

Semiconductor nanoparticles generate radicals in water under irradiation. In the presence of ROS, DTT molecule transforms into its radical form DTT-S• and ultimately results in disulfide molecules ox-DTT (Figures S1 and S2). However, colorless ox-DTT does not react with DTNB to form detectable TNB. Hence, this method can be used to indirectly evaluate radical production. High number of produced radicals results in small quantity of TNB detected and vice versa.

2. Experimental Setup for the Evaluation of Radical Production under Irradiation of 501 nm Visible Light Produced from Nine LEDs

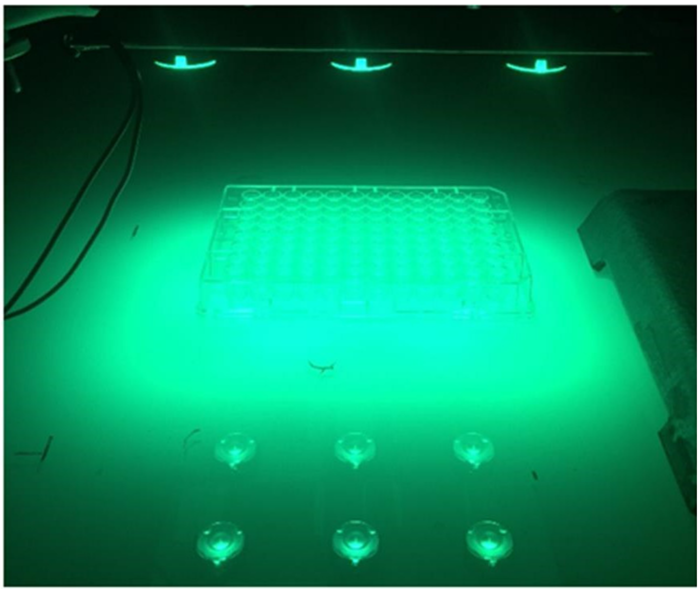

Figure S2. Experimental setup.

3. Complementary Data about ROS Production ex vitro with $Gd_{2(1-x)}Ce_{2x}O_2S$ Nanoparticles

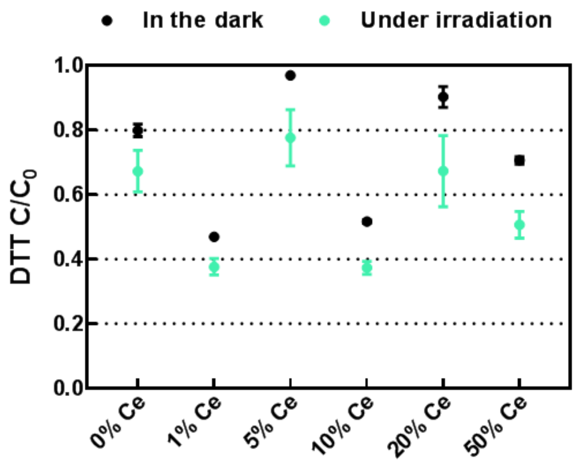

Figure S3. Normalized DTT quantities measured after 4 h in the dark and under irradiation with 1000 µg/mL of $Gd_{2(1-x)}Ce_{2x}O_2S$ nanoparticles (x from 0 to 50%).

4. Specific Surface Area of the Nanoparticles Powders

We investigate the specific surface area of previously studied $Gd_{2(1-x)}Ce_xO_2S$ nanoparticles containing 0, 10 and 50 % of cerium. Nitrogen sorption isotherms were recorded at 77 K with a BEL Japan Belsorp max device, after degassing the powders for 12 h.

Due to the small quantity of sample available, only ca. 10 mg of powder of the nanoparticles was analyzed by adsorption isotherm. The corresponding surface area was deduced using the Brunauer-Emmett-Teller (BET) model. The overall uncertainty on the specific surface is ca 10%.

Table S1. Sodium contents measured by SEM-EDS and specific surface areas measured by adsorption isotherm of $Gd_{2(1-x)}Ce_{2x}O_2S$ nanoparticles with 0, 10 and 50% of cerium. The surface area values were extracted using the Brunauer-Emmett-Teller (BET) theory.

**Sample**	**Na content (eq. vs. lanthanides)**	**$a_{s,BET}$ ($m^2.g^{-1}$)**
0% Ce	0.20 ± 0.03	55
10% Ce	0.33 ± 0.04	81
50% Ce	0.23 ± 0.02	70

The $Gd_{2(1-x)}Ce_xO_2S$ nanoparticles feature specific surface area from 55 to 70 $m^2.g^{-1}$ (**Error! Reference source not found.**). If we consider the intrinsic experimental error of the adsorption isotherm technique (10 $m^2.g^{-1}$) and potential error from the small amount of analyzed powder, there is no clear difference between the samples containing cerium and those that do not. Thus, it is unlikely that this is the origin of the cytotoxicity of the cerium-containing nanoparticles. In order to confirm this, we selected a different $GdCeO_2S$ (50% Ce) sample with lower specific surface area (32 $m^2.g^{-1}$) and evaluated its cytotoxicity by WST-1 assay.

Specific surface of the TiO_2 nanoparticles was 50 m^2/g.

Specific surface of the $BiVO_4$ nanoparticles was 10 m^2/g.

5. Cell Line and Cell Culture

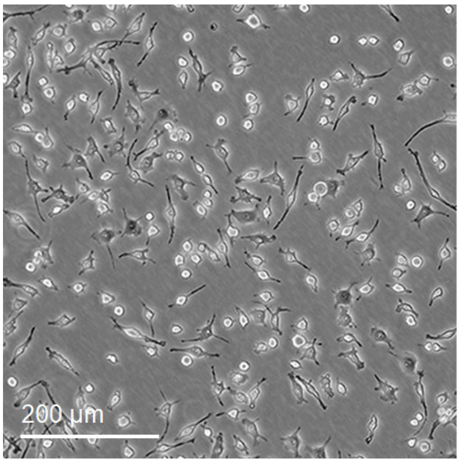

Figure S4. Optical microscope image of murine macrophage RAW 264.7 cell line provided by American Type Culture Collection (ATCC® TIB-71™).

6. Protocol for Measuring the Mitochondrial Activity

WST-1 assay is a colorimetric method to characterize mitochondrial activity of the cells. This method is based on the cleavage of the colorless tetrazolium salt WST-1 to formazan by mitochondrial reductase, which exists in the mitochondrial respiratory chain and is only active in viable cells (Figure S5). An increase of the mitochondrial activity leads to an increase of the production of formazan dye and vice versa. Formazan exhibits a dark yellow color and thus, the mitochondrial activity can be assessed using UV-visible absorption spectroscopy.

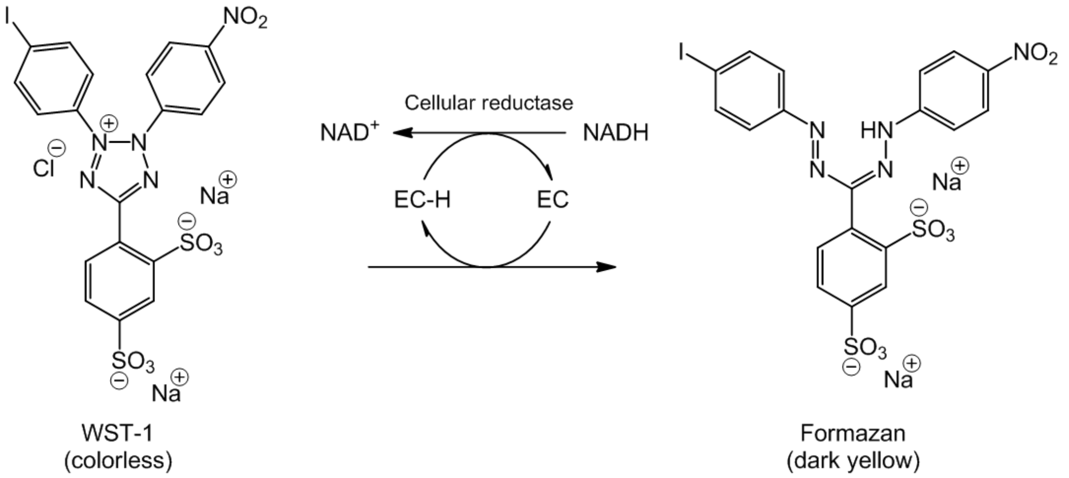

Figure S5. Cleavage of tetrazolium salt WST-1 to formazan by mitochondrial dehydrogenase. EC stands for electron coupling reagent.

Interference test: The decrease of the detected formazan can be due to adsorption of formazan dye to the nanoparticles. In order to verify this, culture medium of RAW 264.7 cells incubated with WST-1 solution for 3 h was taken and mixed with either distilled water as control experiment, or suspensions of nanoparticles for another 3 h. The absorbance at 450 nm was then measured and normalized with that of the control experiment. The results of Gd_2O_2S (0% Ce) and $GdCeO_2S$ (50% Ce) are presented in Figure S6. The normalized absorbance values for both nanoparticle samples (with and without cerium) fluctuate around 1. This indicates that the nanoparticles do not significantly adsorb formazan dye.

The results from the interference test rule out the effect of adsorption of formazan dye by the nanoparticles on our observations.

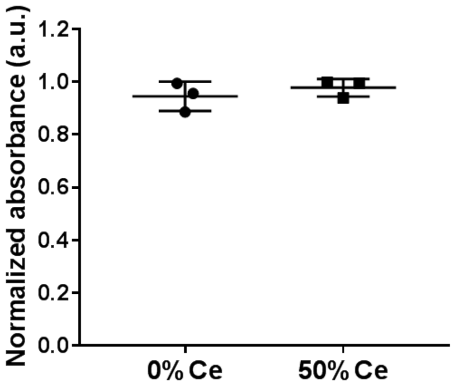

Figure S6. Assay interference test: incubation of formazan dye with 100 µg/mL of $Gd_{2(1-x)}Ce_{2x}O_2S$ nanoparticles. Resulting absorbance is normalized by that of formazan dye.

7. Additional Details about DNA Quantification

Fluorometry is a highly sensitive and simple method to quantify DNA. The principle of this method is essentially based on the binding of fluorescent compounds to nucleic acids by intercalation. One of the most commonly used fluorescent dyes is Hoechst 33258 (Figure S7). When excited at 360 nm, the fluorescence emission at 460 nm of the dye increases significantly in the presence of DNA. This method is capable of detecting nanograms of DNA and can be used to determine cellular DNA content.

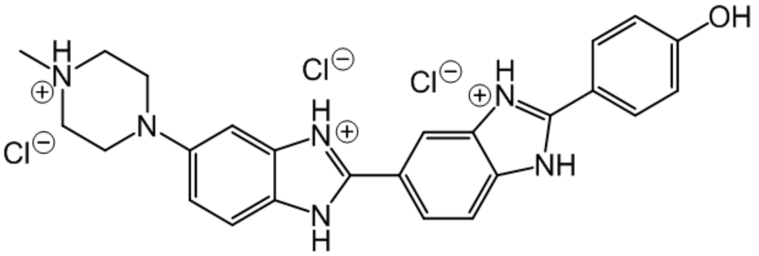

Figure S7. Chemical formula of Hoechst 33258 molecule.

8. Additional Details about Intracellular ROS Activity

The H_2DCF-DA assay is based on fluorescence spectroscopy. This fluorometric assay makes use of an originally non-fluorescent derivative of fluorescein diacetate H_2DCF-DA (Figure S8). This lipophilic molecule can pass through the plasma membrane of the cells and then is de-esterified by non-specific cellular esterase to form non-fluorescent dialcohol H_2DCF. This dialcohol molecule is hydrophilic. Thus, it stays inside the cell and can be eventually oxidized by intracellular ROS to form DCF which is fluorescent. Taking advantage of this process, intracellular ROS production can be measured by simple fluorescence spectroscopy.

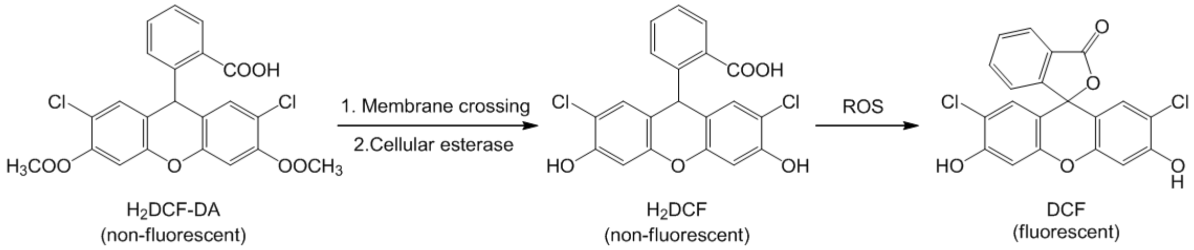

Figure S8. Measuring intracellular ROS activity by H_2DCF-DA assay.

9. X-ray Fluorescence Image of the Control Cells

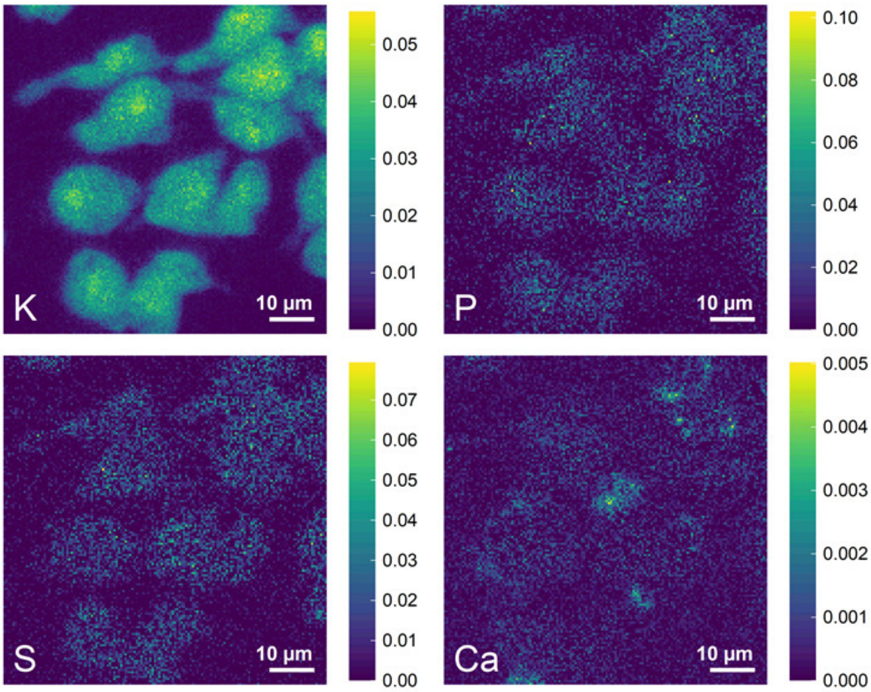

Figure S9. Single-element XRF maps of non-exposed cells showing distribution of K, P, S and Ca. The images are displayed using a linear scale. The values in the color bars represent the concentration of elements in mM. The XRF maps were acquired at 7.4 keV.

References

(1) Nosaka, Y.; Nosaka, A. Y. Generation and Detection of Reactive Oxygen Species in Photocatalysis. *Chem. Rev.* **2017**, *117* (17), 11302–11336. https://doi.org/10.1021/acs.chemrev.7b00161.

(2) Perkins, M. J. Spin Trapping. *Adv. Phys. Org. Chem.* **1980**, *17* (C), 1–64. https://doi.org/10.1016/S0065-3160(08)60127-6.
